# Microalbuminuria among Patients with Diabetes Mellitus Visiting the Department of Nephrology in a Tertiary Care Centre: A Descriptive Cross-sectional Study

**DOI:** 10.31729/jnma.8214

**Published:** 2023-07-30

**Authors:** Tulsi Bhattarai, Asim Pandey, Samriddhi Parajuli, Prajwal Khanal, Angel Dongol, Rahul Devkota, Sohil Neupane, Bharat Kumar Bhattarai

**Affiliations:** 1 Department of Nephrology, National Academy of Medical Sciences, Bir Hospital, Mahaboudha, Kathmandu, Nepal; 2 Department of Cardiology, Norvic International Hospital, Thapathali, Kathmandu, Nepal; 3 Kathmandu Medical College and Teaching Hospital, Sinamangal, Kathmandu, Nepal

**Keywords:** *albumin*, *diabetes mellitus*, *prevalence*

## Abstract

**Introduction:**

Microalbuminuria is an abnormal increase in albumin excretion rate with a specific range of 30-299 mg of albumin/g of creatinine or excretion of 20-200 mg/l of albumin in spot urine samples. Diabetic patients with microalbuminuria are at increased risk for cardiovascular death as compared with normoalbuminuria. The aim of this study was to find out the prevalence of microalbuminuria among patients visiting the Department of Nephrology in a tertiary care centre.

**Method:**

This is a descriptive cross-sectional study conducted among diabetic patients visiting the Department of Nephrology in a tertiary care centre after obtaining ethical approval from the Institutional Review Committee (Reference number: 04072022/04). The study was conducted between 1 October 2022 to 30 November 2022 where patients providing written consent and had documented diabetes were included in the study whereas patients without documented diabetes, having comorbidities such as congestive heart failure, nephritic/nephrotic syndrome and those who refused to give consent were excluded from the study. Convenience sampling was done. Point estimate and 95% Confidence Interval were calculated.

**Results:**

Among 103 patients, microalbuminuria was found to be in 19 (18.45%) (10.96-25.94, 95% Confidence Interval). Out of 19 patients, 8 (42.11%) were male and 11 (57.89%) were female. The mean HbA1c among participants with microalbuminuria was 7.67±0.67%.

**Conclusions:**

The prevalence of microalbuminuria among diabetic patients was similar to other studies done in similar settings.

## INTRODUCTION

Microalbuminuria is an abnormal increase in albumin excretion rate with a specific range of 30-299 mg of albumin/g of creatinine or excretion of 20-200 mg/l of albumin in the spot urine samples.^1^ Diabetic nephropathy leading to renal failure is the second most common cause of death in diabetic patients accounting for one-third of patients with diabetes.^2,3^

Microalbuminuria, a strong predictor of diabetic nephropathy is related to an increased prevalence of systemic hypertension, diabetic retinopathy and neuropathy. Adults with diabetes have a cardiovascular risk that is 2-4 times higher than that of adults without the disease.^3^ Being a silent disease process, it is necessary to determine the prevalence of microalbuminuria in the general population and treat them at the earliest to prevent renal damage and cardiovascular death.

The aim of this study was to find out the prevalence of microalbuminuria among patients with diabetes mellitus visiting the Department of Nephrology in a tertiary care centre.

## METHODS

This was a descriptive cross-sectional study conducted in the Department of Nephrology at Kathmandu Medical College and Teaching Hospital, Sinamangal, Kathmandu, Nepal from 1 October 2022 to 30 November 2022 after taking ethical approval from the Institutional Review Committee (Reference number: 04072022/04). Patients providing written consent and had documented diabetes were included in the study whereas patients without established diabetes, having comorbidities such as congestive heart failure, nephritic/nephrotic syndrome and those who refused to give consent were excluded from the study. Moreover, diabetic patients with comorbid conditions such as congestive heart failure, nephrotic syndrome and athletes participating in excessive exercise were also excluded from the study. Convenience sampling method was done. The sample size was calculated using the following formula:


$$\begin{array}{ll} \text{n} & ={\text{Z}}^{2}\times \frac{\text{p}\times \text{q}}{{\text{e}}^{\text{2}}}\\ & =1.96^{2}\times \frac{0.203\times 0.797}{0.08^{2}}\\ & =98 \end{array}$$

Where,

n = minimum required sample size
Z = 1.96 at 95% Confidence Interval (CI)
p = prevalence of microalbuminuria in diabetic patients taken from previous studies, 20.3%^4^
q = 1-p
e = margin of error, 8%

The minimum required sample size was 98. However, 103 samples were taken for the study.

Patients who were diagnosed with diabetes mellitus and gave consent were worked-up for history and investigation to watch for possible microalbuminuria. After proper written consent, they were subsequently evaluated for the presence of urine microalbumin. Investigations and work-up were done to look for abnormalities of lipid profiles, serum urea, serum creatinine, complete blood count (CBC), arterial blood gas analysis (ABG) and echocardiography. A fundoscopic examination was done to look for diabetic retinopathy. The performed questionnaire was used for the study. Microalbuminuria was diagnosed with a urinary albumin concentration of 20-199 mg/L in a spot urine sample.^1^ Patient demographics and laboratory findings were recorded.

Data were entered in Microsoft Excel and analysis was done using IBM SPSS Statistics version 24.0. Point estimate and 95% CI were calculated.

## RESULTS

**Figure 1:**
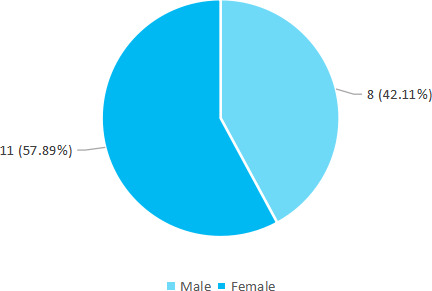
Distribution according to gender (n= 19).

Among 103 patients, microalbuminuria was found to be in 19 (18.45%) (10.96-25.94, 95% CI). Out of 19 patients, 8 (42.11%) were male (Figure 1).

The mean age was 57.45±15.19 years and the mean duration of diabetes was 10.11 ±7.17 years. Mean HbA1c among participants with microalbuminuria was 7.67±0.67%. The mean serum urea concentration among participants with microalbuminuria was 41.58±28.64 mg/dl whereas the mean serum creatinine concentration was 1.38±0.38 mg/dl. Among 19, metformin was used by 7 (36.80%) (Table 1).

**Table 1:** Drugs used (n= 19).

Drugs	n (%)
Metformin	7 (36.80)
Insulin+metformin	5 (26.32)
Metformin+other oral hypoglycemic	7 (36.80)

## DISCUSSION

Among 103 patients, microalbuminuria was found to be in 19 (18.45%). In a similar study, the prevalence of microalbuminuria was found to be 20.3% which was similar to our study. Several non-European populations worldwide have a high prevalence of microalbuminuria, this study confirms and extends previous observations.^5,6^ Furthermore, some studies conducted in India and Nepal reported a higher prevalence of microalbuminuria of 36.3% and 45.5% respectively.^7,8^

In the systematic review, patients with moderately increased albuminuria had a significantly higher risk than those with normoalbuminuria of progressing to severely increased albuminuria.^9^ Other risk factors contributing to the progression to severely increased albuminuria include higher baseline levels of albuminuria, worse glycemic control as estimated from the HbA1c concentration, higher blood pressure, and cigarette smoking.^10-12^

Blood pressure is one of the most important risk factors for microalbuminuria. High blood pressure may cause microalbuminuria by increasing glomerular filtration pressure and subsequent renal damage.^13^ This study showed that 57.9% of patients with high blood pressure had microalbuminuria.

Excess salt intake produces an excess volume load and, thereby, causes an increase in intra-glomerular pressure and glomerular hyperfiltration, which results in the urinary excretion of albumin. An increase in blood pressure is also involved in the mechanism underlying salt-induced albuminuria. An elevated fasting plasma glucose level indicates the presence of insulin resistance that promotes sodium re-absorption and may increase the volume load. Alternatively, endothelial dysfunction may be involved in the mechanism underlying the observed relationship between urinary excretion of albumin and other factors.^14,15^

Although this study is conducted with a sufficient number of participants in a tertiary care centre, there are certain limitations of this study. Laboratory data are obtained from internationally approved laboratory facilities; however, there might be an error in reporting the findings. There might be multiple co-morbidities in a participant that is undiagnosed. This causes an overestimation of the prevalence of microalbuminuria.

## CONCLUSIONS

The prevalence of microalbuminuria in patients diagnosed with diabetes mellitus was found to be similar to other studies done in similar settings.
